# Effect of TSH on Non-Alcoholic Fatty Liver Disease (NAFLD) independent of obesity in children of predominantly Hispanic/Latino ancestry by causal mediation analysis

**DOI:** 10.1371/journal.pone.0234985

**Published:** 2020-06-22

**Authors:** Presley H. Nichols, Yue Pan, Benjamin May, Martina Pavlicova, John C. Rausch, Ali A. Mencin, Vidhu V. Thaker

**Affiliations:** 1 Department of Pediatrics, New York Presbyterian-Columbia University Irving Medical Center, New York, New York, United States of America; 2 Department of Biostatistics, Mailman School of Public Health, Columbia University, New York, New York, United States of America; 3 Herbert Irving Comprehensive Cancer Center, Columbia University Irving Medical Center, New York, New York, United States of America; 4 Department of Pediatrics, Columbia University Irving Medical Center, New York, New York, United States of America; Cincinnati Children's, UNITED STATES

## Abstract

**Background:**

Nonalcoholic Fatty Liver Disease (NAFLD) is a common co-morbidity of obesity. Elevated TSH levels (eTSH), also associated with obesity, may contribute to the dysmetabolic state that predisposes to NAFLD.

**Objective:**

To assess the relationship between TSH levels and NAFLD in children with biopsy-proven NAFLD compared to controls.

**Design and methods:**

In this retrospective study of children with biopsy-proven NAFLD and age-matched controls, the association of eTSH with NAFLD was investigated and the role of TSH as a mediator between obesity and NAFLD was assessed.

**Results:**

Sixty-six cases and 4067 controls (69.7 vs 59% Hispanic/Latino ancestry, p = 0.1) of the same age range seen in the same time duration at an urban Children’s Hospital were studied. Children with NAFLD were more likely to be male (74.6 vs 39.4%, p < 0.001), have higher modified BMI-z scores (median 2.4 (IQR 1.7) vs 1.9 (IQR 1.7), p < 0.001), and abnormal metabolic parameters (TSH, ALT, HDL-C, non-HDL-C, and TG). Multivariate analyses controlling for age, sex and severity of obesity showed significant association between the 4^th^ quartile of TSH and NAFLD. Causal mediation analysis demonstrates that TSH mediates 33.8% of the effect of modified BMI-z score on NAFLD. This comprises of 16.0% (OR = 1.1, p = 0.002) caused by the indirect effect of TSH and its interaction with modified BMI-z, and 17.7% (OR = 1.1, p = 0.05) as an autonomous effect of TSH on NAFLD. Overall, 33.8% of the effect can be eliminated by removing the mediator, TSH (p = 0.001).

**Conclusions:**

The association of eTSH and biopsy-proven NAFLD is demonstrated in children of Hispanic/Latino ancestry. Further, a causal mediation analysis implicates an effect of TSH on NAFLD, independent of obesity.

## Introduction

Non-alcoholic fatty liver disease (NAFLD), defined as hepatic steatosis by imaging or histology without a secondary cause of hepatic fat accumulation [1], has become the most common chronic liver disease in children in parallel with the rising prevalence of obesity [2]. NAFLD is considered the hepatic manifestation of the metabolic syndrome which includes high blood pressure, dyslipidemia, insulin resistance and truncal obesity [3]. The spectrum of NAFLD includes simple steatosis through steatohepatitis. Simple steatosis may have a benign course, but steatohepatitis can lead to cirrhosis or hepatocellular carcinoma [4]. The gold standard for the diagnosis and staging of steatohepatitis is liver biopsy.

Thyroid hormone is an important regulator of hepatic lipid metabolism through induction of genes involved in hepatic lipogenesis, coupling of autophagy to mitochondrial fat oxidation thereby leading to ketogenesis, and causing reverse cholesterol transport [5]. Therefore, it is not surprising that a close relationship has been observed between elevated TSH levels (eTSH) and cardiometabolic risk factors and NAFLD in adults [6–8]. A small number of studies in Caucasian children, primarily from Europe, have shown the association between eTSH and NAFLD defined by hepatic ultrasound in children and adolescents with obesity [9–12]. Animal experiments have demonstrated that the TSH receptor is expressed in hepatocytes [13] and that TSH may have an essential role in the mitochondrial stress in the liver involved in NAFLD [14].

The prevalence of NAFLD is higher in older males, especially those of Hispanic/Latino origin [15, 16], however, little is known about the association of NAFLD and eTSH in this population. The goal of this study was to identify the relationship between eTSH and NAFLD in a cohort of children of predominantly Hispanic/Latino ancestry with biopsy-proven NAFLD. Further, given the role of thyroid hormones in lipid metabolism and NAFLD, we hypothesized that TSH may be a causal mediator for the development of NAFLD in children with obesity.

## Materials and methods

### Cohort selection

In this retrospective study, the cases were identified from a registry of children with biopsy-proven NAFLD from the Pediatric Gastroenterology and Hepatology program at Columbia University Irving Medical Center (CUIMC) between 2010–2018. Children with persistently elevated serum aminotransferase (ALT/AST) level with or without clinical suspicion of NAFLD are routinely evaluated for infectious, autoimmune and heavy metal exposure as the etiology, along with an assessment of thyroid function. Children who continued to have persistently elevated ALT/AST levels despite lifestyle interventions and no known etiology underwent liver biopsy [1, 4] to confirm the diagnosis as well as to assess inflammation, fibrosis and the degree of hepatic fat deposition.

The control subjects were children of the age range of the cases attending the primary care clinics (2010–2018) at the same institution with available TSH and free T4/total T4 levels and normal liver function tests (LFTs) defined by the laboratory reference range and absence of any acute or chronic liver disease by ICD-codes. Elevated ALT is considered a biomarker for NAFLD [4], hence only children with ALT ≤ 45 IU/L were considered as controls [4]. Retrospective lifetime data were collected from the electronic health records (EHRs) including prior physical examinations, anthropometric parameters measured during routine clinical care, laboratory tests, imaging studies and diagnoses codes. All subjects with ICD-codes consistent with renal disease, diabetes, known thyroid or other endocrine disease were excluded. Body mass index (BMI) was calculated as weight in kg/(height in m)^2^. BMI-z scores and modified BMI-z (mod-BMI-z) were calculated using the LMS parameters from CDC 2000 growth charts [17, 18] [URL]. Mod-BMI-z has been shown to be a better measure of adiposity than BMI-z in children with severe obesity [17] [URL]. BMI class was considered normal BMI between 5-85^th^ percentile for age, moderate obesity as BMI between the 85^th^ percentile-120% of 95^th^ percentile, and severe obesity as BMI > 120% of 95^th^ percentile for age [19]. eTSH was defined as TSH levels in the 4^th^ quartile for the population under study and subclinical hypothyroidism (SH) as TSH levels > 4.5 mIU/L. The population served by CUIMC is primarily children residing in the Washington Heights neighborhood of New York City where the racial/ethnic background is 71% Hispanic/Latino, 7% non-Hispanic Black, 17% non-Hispanic Whites, 3% Asian and 1% others according to the U.S. Census Bureau Population estimates, 2013. The ethnicity of the study population was mapped by using the data entered in the “Ethnicity” field of the EHRs. For the individuals where the ethnicity data was missing, primary language reported as Spanish was used to stratify the individual as Hispanic/Latino. The resulting racial/ethnic distribution of the cohort was compared to the race/ethnicity distribution in the zip codes of primary residence of the subjects included in the cohort.

### Liver biopsy

Liver biopsy was performed in patients with evidence of hepatic steatosis on imaging with excluded secondary causes of liver disease and persistently elevated aminotransferases despite lifestyle interventions as per the standard of care guidelines [1, 4]. Needle biopsy was performed in a pediatric procedure unit with anesthesia. Biopsy specimen was sent to the Columbia University Pathology Department for histological analysis of liver inflammation, fibrosis and fat deposition based on hematoxylin and eosin staining as well as trichrome staining when applicable. As per pathology guidelines, NAFLD was defined as macrovesicular steatosis in greater than 5% of hepatocytes or presence of Non-Alcoholic Steato-Hepatitis (NASH) [20].

### Laboratory measurements

Results of laboratory tests were ascertained from the EHRs. Serum concentrations of total cholesterol (TC), high-density lipoprotein cholesterol (HDL-C) and triglycerides (TG) were measured using routine enzymatic methods with Roche/Hitachi Cobas® c system analyzer (USA). Value of low-density cholesterol (LDL-C) was calculated using Friedwald equation. Standard liver function tests, including ALT, AST, alkaline phosphatase and total bilirubin were measured on the same day with an auto analyzer. Non-HDL cholesterol was calculated as TC minus HDL-C. Serum samples were assayed for fT4, total T4 and TSH levels using an automated chemiluminescence assay system on Roche e601 platform. Peak values for TSH, ALT, TG, HDL-C, LDL-C and non-HDL cholesterol were extracted from individuals’ lab test results. Subjects were excluded if they had TSH value greater than 10 mIU/L or positive antithyroid antibodies and/or received thyroid related medications.

### Statistical methods

A descriptive analysis of the cohort was performed by calculating absolute and relative proportions for categorical variables, and the mean and standard deviation for continuous variables. Distribution of continuous variables were examined for skewness and kurtosis and were logarithmically transformed, when appropriate. Median and interquartile range as well as the data range were used for summary statistics of each group in the cohort. To assess differences between the subjects with and without NAFLD, the Wilcoxon rank-sum test was applied for continuous variables and the chi-squared test for categorical variables. TSH values were categorized into four quartiles for the univariate and multivariate analysis. Univariate analysis was performed to assess the association between TSH quartiles and NAFLD. Multivariate logistic regression was used to determine the association between TSH quartiles and NAFLD adjusted for age, gender and severity of obesity. Similar methods were used for association between the individual lipid levels and TSH. For sensitivity analyses, the above analyses were also performed with TSH as a binary variable using a level > 4.5 mIU/L considered as SH.

To explore causal mediation of TSH levels, mediation analysis using the counterfactual framework was performed between mod-BMI-z (obesity) and the presence of NAFLD (binary outcome) with TSH as the mediator using logit link along with exposure mediator interaction [21, 22]. Age and gender were used as covariates in the causal mediation models. The mediation effect was decomposed into 4-components: i) direct effect: the effect of the exposure (mod-BMI-z) on the outcome (NAFLD) in the absence of the mediator (TSH); ii) reference interaction effect: the effect of the mediator (TSH) on the outcome (NAFLD) in the absence of the exposure (mod-BMI-z); iii) a mediated interaction: the interaction of exposure (mod-BMI-z) and the mediator (TSH); and iv) pure mediated effect: mediation of TSH between the exposure and the outcome [23]. TSH levels were log-transformed and centered for these analyses. The analyses were performed using SAS v3.7 (the CAUSALMED procedure, SAS Institute, Cary, NC, USA) and R-statistical software v3.2 [URL] using libraries aov, dplyr, DescTools, lmtest, MKmisc and ggplot2. A two tailed p-value < 0.05 was considered significant. The study protocol was approved by the Institutional Review Board (IRB) at Columbia University Medical Center. The IRB waived the informed consent requirement due to the observational retrospective nature of the study, and a coded study ID was assigned to all the subjects prior to analysis. All data were stored and data analysis was undertaken on password protected encrypted institutional servers.

## Results

From the registry of 78 children with biopsy-proven NAFLD, 66 subjects with available thyroid studies were included as cases. There were no differences in the age, gender, BMI or lipoprotein profile of the excluded cases. Of the total 21,258 unique children in the same age range seen at the primary care clinic in the same time period, 4300 subjects had data available on thyroid function tests and lipoproteins variables. One hundred eighty-three subjects were eliminated for elevated liver enzymes, and 50 for biologically implausible anthropometric values or chronic disease based on ICD-codes including thyroid disease. The reasons for requesting TSH levels included obesity, growth delay, fatigue, changes in hair, skin or nails or family request. The final study cohort comprised of 66 cases with biopsy proven NAFLD and 4067 controls in the same age range. The demographic details of the cohort are provided in Table 1. Briefly, there was a higher number of males in the NAFLD group. The BMI z-score, mod-BMI-z and the proportion of individuals with severe obesity was higher in the NAFLD group compared to the controls. All metabolic parameters assessed in the study (TSH, ALT, HDL-C, non-HDL-C, LDL-C and TG) were significantly higher in the cases with NAFLD, and free T4 levels were similar (Table 1). The high ALT levels of the cases are consistent with the expected presence of NASH [24].

**Table 1:** Demographic distribution of the cases and controls.

Parameter	n	Subjects with NAFLD	Subjects without NAFLD	p-value
	(Cases,	Median (IQR)	Median (IQR)	
	Controls)	[range]	[range]	
Gender, n (%)	(66,			≤0.0001
Male	4067)	47 (74.6%)	1561 (38.3%)	
Age (yrs)	(66,	12.7 (10.2)	16 (12.3)	
	4067)	[5.6–18.7]	[5.4–18.8]	
Ethnicity				0.3
Hispanic	(66,	46 (69.7%)	2401 (59.0%)	
Non-Hispanic	4067)	5 (7.6%)	338 (8.3%)	
Unknown		16 (22.7%)	1328 (32.7%)	
BMI-z score	(66,	2.1 (0.7)	1.2 (1.8)	≤0.0001
	4067)	[1.0–3.0]	[-2.6–3.6]	
Mod BMI z-score	(66,	2.4 (1.7)	1.9 (1.7)	
	4067)	[0.8–5.1]	[-2.4–12.2]	
BMI class*, n(%)				
Normal weight	(66,	9 (13.6%)	2621 (64.4%)	
Mild obesity	4067)	26 (39.4%)	788 (19.4%)	
Severe obesity		31 (46.9%)	658 (16.2%)	
TSH	(66,	2.4 (1.9)	1.6 (1.3)	
(mIU/L)	4067)	[0.5–8.4]	[0.01–9.7]	
ALT	(66,	95.5 (65.5)	13 (8.0)	
(U/L)	4067)	[28.0–427.0]	[3.0–45.0]	
Non-HDL-C	(65,	125.0 (107.0)	106.0 (88.0)	
(mg/dL)	2481)	[45.0–216.0]	[27.0–332.0]	
HDL-C	(65,	42.0 (35.0)	55.0 (16.0)	
(mg/dL)	2481)	[28.0–65.0]	[9.0–181.0]	
TG	(66,	148.0 (109.0)	92.0 (67.0)	
(mg/dL)	2481)	[47.0–312.0]	[22.0–2016.0]	
LDL-C	(65,	92.0 (78.0)	85 (33)	0.06
(mg/dL)	2481)	[34.0–187.0]	[13.0–262.0]	
free T4	(40,	1.1 (0.2)	1.1 (0.3)	0.6
(ng/dL)	3937)	[0.9–1.4]	[0.4–4.7]	

* Normal weight = <85th BMI ercentile for age/sex

Mild obesity = 85–120% of 95th BMI percentile for age/sex

Severe obesity = > 120% of 95th BMI percentile for age/sex

**Fig 1:**
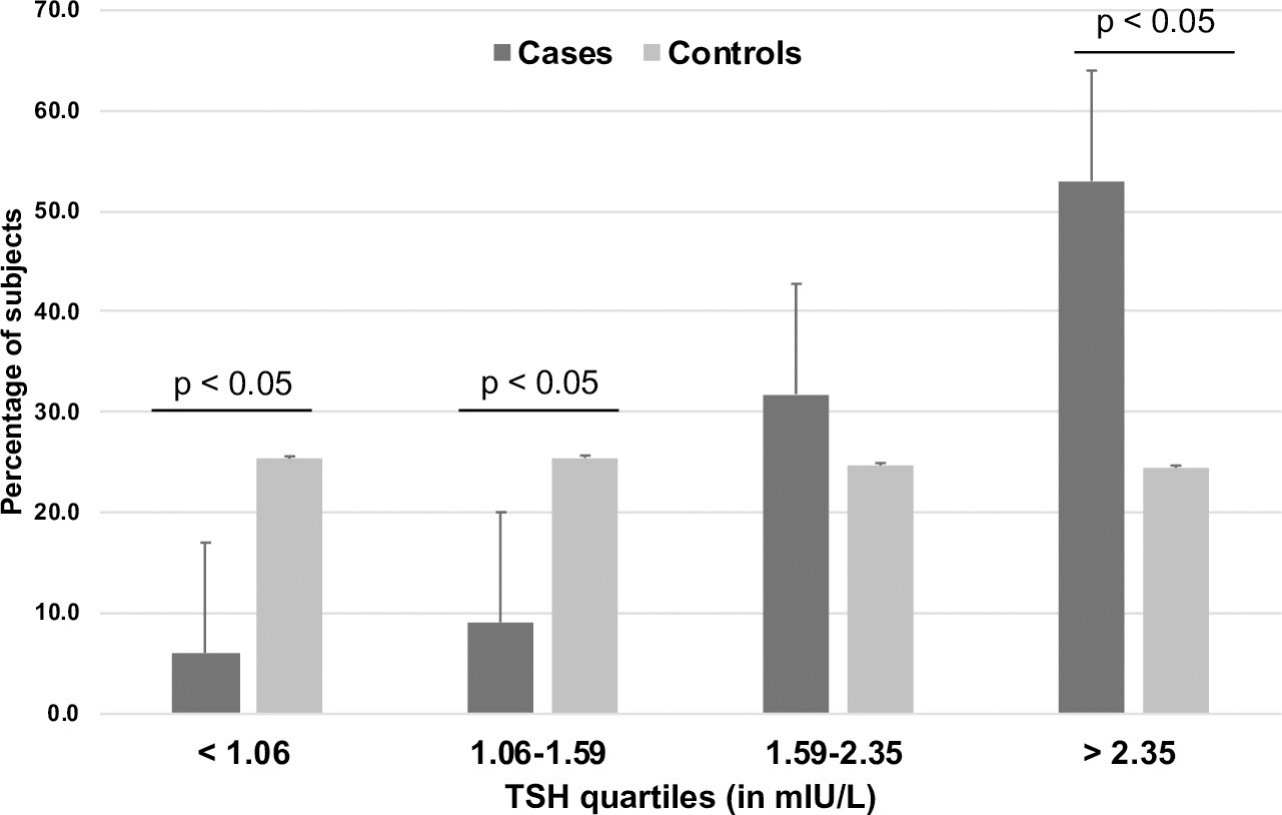
Percentage of cases and controls in each TSH quartile. There were lower number of cases compared to controls in the 1^st^ and 2^nd^ quartiles (p < 0.05) and higher number of cases compared to controls in the 4^th^ quartile of TSH level (p < 0.05).

The TSH levels were divided into 4 quartiles: TSH Q1 < 1.06, TSH Q2 1.06–1.59, TSH Q3 1.59–2.35, TSH Q4 > 2.35 mIU/L (Table 2). In the univariate analyses there were higher odds of the presence of NAFLD in the 3^rd^ and 4^th^ quartile of TSH compared to TSH Q1 (n = 1036, treated as reference). Controlling for age, gender, and class of obesity, this association reached statistical significance in the 4^th^ quartile. ALT, non-HDL-C, and triglycerides showed a similar pattern but not LDL-C, HDL-C and TC (Table 2). While the total number of children across the 4 quartiles are similar (Table 2), there is a higher proportion of cases in the 4th quartile of TSH compared to the controls (Fig 1).

**Table 2:** Association between TSH and NAFLD, ALT and lipoproteins.

TSH quartile	TSH Q2		TSH Q3		TSH Q4	
	(n = 1041)		(n = 1026)		(n = 1030)	
TSH range (mIU/L)	1.06–1.59		1.59–2.35		> 2.35	
Multivariate regression	OR*	p-value	OR*	p-value	OR*	p-value
	(95% CI)		(95% CI)		(95% CI)	
NAFLD	0.9	0.9	2.9	0.05	4.6	**0.005**
	(0.3–3.9)		(1.1–10.4)		(1.8–15.8)	
ALT	0.8	0.6	1.6	0.2	2.5	**0.007**
	(0.4–1.8)		(0.80–3.24)		(1.3–5.0)	
non HDL-C	1.1	0.5	1.3	0.06	1.5	**0.002**
	(0.8–1.4)		(0.9–1.7)		(1.2–1.9)	
Triglycerides	0.9	0.8	1.3	**0.03**	1.4	**0.007**
	(0.8–1.6)		(1.0–1.6)		(1.1–1.8)	
LDL-C	0.9	0.6	1	0.9	1.2	0.2
	(0.7–1.2)		(0.8–1.4)		(0.9–1.7)	
HDL-C	1	0.9	0.8	0.2	0.8	0.1
	(0.8–1.3)		(0.7–1.1)		(0.7–1.1)	
Total Cholesterol	1	0.9	0.9	0.9	1.2	0.1
	(0.8–1.3)		(0.8–1.3)		(0.9–1.6)	

Q = quartile

Normal range used: ALT: < 30 mIU/L; non HDL-C: < 125 mg/dL (3.24 mmol/L)

Triglycerides: 0–9 yrs < 75 mg/dL (0.85 mmol/L); 10–19 yrs < 90 mg/dL (1.02 mmol/L); LDL-C: < 110 mg/dL (2.85mmol/L)

HDL-C: < 45 mg/dL (1.17 mmol/L); Total Cholesterol < 170 mg/dL (4.40 mmol/L).

TSH Q1 (TSH < 1.06 mIU/L) was used as a reference for the OR.

*Covariates for analysis = age, sex and obesity class

Sensitivity analyses with a TSH level of > 4.5 mIU/L, considered the upper limit of normal by many labs, controlling for age, gender and BMI class showed a significant association with NAFLD (OR = 3.5 [95% CI 1.6,7.2], p = 0.001) that persisted while controlling for non-HDL-C (OR = 3.3 [95% CI 1.4,6.8], p = 0.002). No association was noted between abnormal TSH category and LDL-C, HDL-C, non-HDL-C, TC or TG with binary classification of TSH. Likelihood ratio test and Hosmer-Lemeshow test showed good fit of the logistic regression models.

The causal mediation analysis showed 66.2% (OR = 1.4 [95% CI 1.2,1.7], p < 0.0001) direct effect of mod-BMI-z on the presence of NAFLD. The mediated effect of TSH on NAFLD was 16.0% (OR = 1.1 [95% CI 1.0–1.1], p = 0.002) comprising of 10.4% of pure mediation and 5.6% is the interaction between mod-BMI-z and TSH. Interestingly, 17.7% (OR = 1.1 [95% CI 1.0–1.2], p = 0.05) of the effect was the reference interaction caused by TSH on NAFLD, without the influence of mod-BMI-z (Table 3). The total interaction effect of TSH, both from its interaction with mod-BMI-z and the independent effect, was 23.3% (p = 0.02). Of the total effect of mod-BMI-z on NAFLD, 33.8% can be eliminated by intervention on the mediator, TSH.

**Table 3:** Causal mediation analysis of TSH as a mediator of mod BMIz and NAFLD.

**a. Summary of Effects**			
** **	**Estimate**	**95% CI**	**p-value**
Odds Ratio Total Effect	1.6	(1.3–1.8)	< 0.0001
Odds Ratio Controlled Direct Effect (CDE, mod BMIz on NAFLD)	1.4	(1.2–1.7)	< 0.0001
Odds Ratio Natural Indirect Effect (NIE, mod BMIz acting through TSH)	1.1	(1.0–1.1)	0.0001
Total Excess Relative Risk	0.6	(0.4–0.8)	< 0.0001
Excess Relative Risk due to CDE	0.4	(0.2–0.6)	< 0.0001
Excess Relative Risk due to NIE	0.1	(0.04–0.1)	0.0002
**b. Four-way decomposition of mediated effect (percentage)**			
Direct Effect of mod BMIz on NAFLD	66.2	(45.5–86.9)	< 0.0001
Effect of TSH independent of mod BMIz	17.7	(1.6–33.9)	0.03
Effect of interaction b/w TSH and mod BMIz	5.6	(2.7–8.5)	0.0002
Pure mediation via TSH	10.4	(3.8–17.1)	0.002
Total mediation via TSH	16.0	(8.4–23.7)	< 0.0001
Percentage Eliminated	33.8	(13.0–54.5)	0.001

## Discussion

To our knowledge, this is the first study to examine the association and causal mediation of TSH levels with biopsy-proven NAFLD with controls in children of predominantly Hispanic/Latino ancestry. The causal mediation analysis demonstrates a partial mediation role of TSH in the relationship between the mod-BMI-z score and the presence of NAFLD. The 4-way decomposition of the causal mediation model shows an effect of elevated TSH on NAFLD independent of mod-BMI-z. Collectively, these findings provide evidence for a mechanistic role of elevated TSH levels in the association between mod-BMI-z and NAFLD.

TSH levels between 4.5–10 mIU/L that do not reach the threshold for treatment, also called subclinical hypothyroidism (SH), is a common occurrence in adults with a tendency towards development of overt hypothyroidism [25]. SH has been variably associated with adverse cardiometabolic effects, including dyslipidemia, insulin resistance, diastolic dysfunction, endothelial dysfunction, coronary heart disease and heart failure in adults [6, 26, 27]. On the other hand, SH among children has been considered a benign and remitting condition, with the risk for progression only seen in individuals with certain underlying causes, such as autoimmune disease [28]. The evidence for the effect of SH on the growth and neurocognitive outcomes is conflicting [29, 30], but the evidence for the proatherogenic and metabolic abnormalities is being increasingly recognized [31–35].

In this study, the children with NAFLD had a higher degree of obesity (BMI-z, mod-BMI-z and proportion of children with severe obesity) with higher levels of non-HDL-C and TG. This is consistent with published reports. In a retrospective cross-sectional analysis of 528 euthyroid children, Aypak *et al* found an association of higher levels of TSH and fT3 levels with obesity, and an association of increased level of dyslipidemia and higher systolic blood pressure with higher levels of TSH [31]. In a cohort of 49 prepubertal children with SH for a minimum of 2 years and age matched controls, Cerbone *et al* found higher cardiometabolic risk factors, measured in the form of waist to height ratio, atherogenic index (ratio of TC: HDL-C), TG: HDL-C ratio, homocysteine levels, and lower HDL-C levels [32]. These findings were replicated in a cohort of adolescents from the community-based Korea National Health and Nutrition Examination surveys [35]. Similar observations were made in a cohort of 22,147 children with Type 1 diabetes collected from multiple centers across Germany with or without the presence of autoantibodies [34]. Pacifico *et al* extended the observations of the association of metabolic findings with SH to NAFLD. Their study of 49 children with NAFLD and matched controls showed that in addition to the association with hypertriglyceridemia and insulin resistance, SH (TSH > 4.5 mIU/L) was associated with NAFLD, adjusting for age, gender, BMI and free T3/T4 levels [11]. In a separate study, higher insulin resistance, carotid intimal thickness and left ventricular mass were found in children and adolescents with SH and NAFLD compared to matched obese and lean controls [36].

Since many of the human studies on SH have been performed in children with obesity, there is debate about the directionality [30]. The inflammatory state in obesity is thought to influence the sodium-iodine symporter causing hyperthyrotropemia that may resolve with weight loss [37]. Animal models have indicated that hyperthyropinemia seen with obesity may be driven by the enhanced leptin-mediated production of pro-thyrotropin releasing hormone (TRH) [38] or due to impaired feedback due to decreased number of T3 receptors in the hypothalamus [39]. It is unknown whether this is true in humans or whether hyperthyrotropinemia, dyslipidemia and NAFLD are driven by similar (or serial) mechanisms and in what direction. The mediation analysis performed in this study attempts to address this question. Given the known biology of the influence of thyroid hormone on lipid metabolism [5], we hypothesized that TSH is a mediator of the effect of obesity on NAFLD. The causal mediation analysis under the counterfactual framework showed that the association between obesity and NAFLD is partially mediated by TSH. The more interesting finding from this analysis was the identification of the effect of TSH on NAFLD independent of the mod-BMI-z thereby raising the question of whether children with elevated TSH in the absence of obesity are also at a higher cardiometabolic risk. While studies of children with long-term untreated autoimmune and idiopathic SH do not show higher overall adiposity, they may have higher visceral adiposity [40]. Indeed, in two prior studies, waist circumference and waist-to-height ratio were higher among children with mild SH than among healthy euthyroid children matched for age, gender, height and pubertal status [40, 41].

Thyroid hormones have direct effects on both cholesterol and fatty acid synthesis and metabolism in a cell autonomous manner by transcriptional regulation of target genes involved in several of the hepatic metabolism pathways. Animal studies have shown that mildly hypothyroid mice develop NAFLD by impaired suppression of adipose tissue lipolysis as well as upregulation of de novo lipogenesis due to breakdown in the complex metabolic pathways in the liver [42]. Aside from this, TSH has been independently found to modulate lipid metabolism through the TSH receptors on the hepatocytes to induce hepatosteatosis via sterol regulatory element binding protein, SREBP [43]. TSH also suppresses the synthesis of hepatic bile acid via an SREBP2-hepatocyte nuclear factor 4α (HNFα)- CYP7A1 signaling pathway [44]. Moreover, TSH inhibits cholesterol synthesis by increasing AMPK-mediated phosphorylation of hydroxymethylglutaryl-CoA reductase (HMGCR) to inhibit HMGCR activity [45]. Collectively, these findings support the independent role of TSH in the regulation of both hepatic lipid and cholesterol homeostasis.

The strengths of this study are the availability of a cohort of children with NAFLD proven by liver biospy, and the matched control group that allowed for a causal mediation analysis in a population of children of predominantly Hispanic/Latino ancestry. The limitations are the retrospective nature of the cohort, and data extraction from EHRs that precludes confirmation by repeating the tests or performing additional ones. It is possible that some of the controls, especially those with obesity, have undiagnosed NAFLD and/or SH associated with obesity. If this is the case, the results of this study perhaps represent the tip of the iceberg and may be magnified in a prospective well-controlled study. We were unable to include measures such as race, glucose, insulin or leptin levels, blood pressure, waist circumference or pubertal status in this analysis because either the data were incomplete or the accuracy of ascertainment could not be assured. We included the ethnicity data from EHRs that was concordant with the census data from the zipcodes where the patients resides. It is possible that some additional fraction of subjects labeled “Unknown” in the EHRs may be of Hispanic/Latino ancestry, and the method of ascertainment underestimates the proportion. Further, while we used the thyroid function tests obtained prior to the established diagnosis of NAFLD, it is possible that undetected NAFLD was already present at the time, in which case, the relationship is an association rather than mediation. TSH levels may fluctuate in health and disease and while these variations can be overcome at a cohort level, prospective studies with repeated measurements of TSH levels are needed before these results can be used in practice for individual patients. Regardless, at a group level, the data captures the natural variability of TSH at population level and the results of this study make an important contribution to the growing body of evidence on the complex relationship between thyroid hormone, lipid metabolism and its role in long-term cardiometabolic consequences of obesity and its associated long-term health outcomes. The results of this study can be considered the preliminary data to spark further conversation and longitudinal studies to understand these mechanisms in humans towards more effective clinical management strategies in future.
